# Membranes Prepared from Recombinant RGD-Silk Fibroin as Substrates for Human Corneal Cells

**DOI:** 10.3390/molecules26226810

**Published:** 2021-11-11

**Authors:** Elham Nili, Damien G. Harkin, Rebecca A. Dawson, Neil A. Richardson, Shuko Suzuki, Traian V. Chirila

**Affiliations:** 1 School of Biomedical Sciences, Queensland University of Technology, Brisbane, QLD 4000, Australia; elham_nili64@yahoo.com (E.N.); d.harkin@qut.edu.au (D.G.H.); rebdawson@outlook.com (R.A.D.); nar.richardson@outlook.com (N.A.R.); 2 Queensland Eye Institute, South Brisbane, QLD 4101, Australia; shuko.suzuki@qei.org.au; 3 School of Chemistry & Physics, Queensland University of Technology, Brisbane, QLD 4000, Australia; 4 Australian Institute of Bioengineering & Nanotechnology, University of Queensland, St. Lucia, QLD 4072, Australia; 5 Faculty of Medicine, University of Queensland, Herston, QLD 4006, Australia; 6 School of Molecular Science, University of Western Australia, Crawley, WA 6009, Australia; 7 Faculty of Medicine, George E. Palade University of Medicine, Pharmacy, Science & Technology, 540139 Târgu Mureş, Romania

**Keywords:** silk fibroin, RGD peptides, corneal cells, cell adhesion, cell culture

## Abstract

A recombinant formulation of silk fibroin containing the arginine–glycine–aspartic acid (RGD) cell-binding motif (RGD-fibroin) offers potential advantages for the cultivation of corneal cells. Thus, we investigated the growth of corneal stromal cells and epithelial cells on surfaces created from RGD-fibroin, in comparison to the naturally occurring *Bombyx mori* silk fibroin. The attachment of cells was compared in the presence or absence of serum over a 90 min period and analyzed by quantification of dsDNA content. Stratification of epithelial cells on freestanding membranes was examined by confocal fluorescence microscopy and optimized through use of low molecular weight poly(ethylene glycol) (PEG; 300 Da) as a porogen, the enzyme horseradish peroxidase (HRP) as a crosslinking agent, and stromal cells grown on the opposing membrane surface. The RGD-fibroin reduced the tendency of stromal cell cultures to form clumps and encouraged the stratification of epithelial cells. PEG used in conjunction with HRP supported the fabrication of more permeable freestanding RGD-fibroin membranes, that provide an effective scaffold for stromal–epithelial co-cultures. Our studies encourage the use of RGD-fibroin for corneal cell culture. Further studies are required to confirm if the benefits of this formulation are due to changes in the expression of integrins, components of the extracellular matrix, or other events at the transcriptional level.

## 1. Introduction

Fibroin is the protein responsible for the mechanical properties of silk fibers and can be readily isolated from the cocoons of different Lepidopteran species, mainly from those produced by the domesticated silkworm *Bombyx mori*. This fibrous protein (henceforth, BMSF) is a biocompatible and biodegradable material which, owing to its remarkable physicochemical properties, has been employed as a biomaterial in a variety of tissue engineering and biomedical applications [1,2,3,4,5,6,7]. As a polymeric material, BMSF is a composite of naturally designed block copolymers. As a protein composite, BMSF consists of a heavy-chain fibroin (~360–390 kDa) linked through disulfide bridges to a light-chain fibroin (~25 kDa), and a glycoprotein (fibrohexamerin, ~30 kDa) that is associated non-covalently [8,9,10]. The techniques employed to isolate fibroin from the cocoon material lead to a mixture of polypeptides derived via hydrolysis of the native proteins. A range of morphologies can be readily fabricated from aqueous solutions of fibroin including porous sponges, electrospun fibers, thin films, coatings, and freestanding membranes, which display potential value as scaffolds for tissue regeneration or vehicles for cell transplantation. In particular, the BMSF templates have been explored as a material for repairing the ocular surface, based on their high transparency and greater stability than the membranes prepared from other biomaterials [2,5,11,12,13,14].

Chirila et al. [11] were the first to use BMSF membranes for reconstructing the ocular surface, demonstrating the attachment and growth of corneal epithelial cells derived from progenitor cells isolated from the corneal limbus. Subsequent studies have confirmed the additional suitability of fibroin membranes as a substrate for corneal stromal cells [12,13] and corneal endothelial cells [14]. Thus, cells derived from all three layers of the cornea display capacity for growth on silk fibroin. Nevertheless, in the absence of naturally occurring cell-adhesion motifs, attachment of cells to BMSF is primarily dependent upon the use of serum-supplemented growth media and often requires further optimization by coating with purified extracellular matrix (ECM) components such as collagens or vitronectin [14,15,16]. As an alternative to this approach, a number of studies [17,18] have explored altering the adhesive properties of fibroin via incorporation of the classical cell-binding motif abbreviated to RGD (arginine–glycine–aspartic acid). Studies by Gil et al. [18] for example, have demonstrated faster growth of a virally transformed corneal fibroblast cell line when using fibroin membranes that had been chemically bound to an exogenous peptide containing the RGD motif. In contrast, we have reported this strategy to be ineffective for enhancing the attachment of primary corneal epithelial cells to fibroin [17]. As an alternative approach, we have previously attempted to exploit the presence of naturally occurring RGD sequences that is present in fibroin isolated from the wild silkworm *Antheraea pernyi* (APSF) [17,19]. Nevertheless, the freestanding membranes made of APSF were found to be technically more difficult to produce than those derived from BMSF [19], and no benefits were observed when blends of BMSF and APSF were used as substrates for primary corneal epithelial cells [17]. An alternative option, however, that has yet to be investigated for corneal cells, is to use fibroin that has been genetically engineered to contain RGD peptide sequences [20,21].

In order to produce fibroin containing the RGD sequence, Kambe et al. [20,21] have genetically engineered *Bombyx mori* silkworms to produce fibroin light chains fused directly to two sequential RGDS sequences (L-RGDS × 2 or LRF, in the authors’ terminology). Chondrocytes seeded onto films cast from the RGD-fibroin adhered with enhanced force when compared to those attached to non-modified fibroin films during the initial stages after seeding [20]. Subsequent analyses revealed increased expression of cell-adhesion molecules (integrins) and the ECM molecule aggrecan over the same period of time. These findings suggest that the recombinant formulation of fibroin as developed by this group may provide a superior substrate for cell transplantation, especially when repairing tissues are exposed to external frictional forces such as in the ocular surface.

We therefore sought presently to evaluate the response of epithelial cells and stromal cells derived from the human corneal limbus to the substrates prepared from recombinant RGD-fibroin. We commence by examining the short-term (90 min) response of both cell types to tissue culture plastic coated with RGD-fibroin and subsequently examine the longer-term growth response of stromal cells and epithelial cells to cast films and freestanding membranes, respectively. The freestanding membranes are subsequently modified utilising low molecular weight poly(ethylene glycol) (PEG; MW 300 Da) as a porogen [22], in an effort to enhance the epithelial stratification via diffusion of soluble factors derived from stromal cells grown on the opposing membrane surface. Moreover, an attempt is made to strengthen the PEG-treated RGD-fibroin membranes through use of horseradish peroxidase (HRP) as an enzymatic crosslinking agent [23]. Our results demonstrate an altered response of both cell types to the genetically modified formulation of fibroin, with these responses becoming more apparent in longer-term cultures. We also demonstrate enhanced mechanical properties and permeability of freestanding membranes as a result of undergoing the treatments with PEG and HRP.

## 2. Results

### 2.1. Short-Term Response of Limbal Cells to RGD-Fibroin

#### 2.1.1. Attachment of Limbal Stromal Cells

The relative attachment of limbal stromal cells to standard fibroin (BMSF), compared to a recombinant formulation of fibroin incorporating the RGD cell-binding motif, was assessed both visually and by quantification of dsDNA as a proxy for cell numbers. For the purpose of these cell binding studies, each formulation of fibroin was applied as a coated film on tissue culture plastic (TCP). Non-coated TCP was used as a positive control and all tests were performed over 90 min in the presence of 10% foetal bovine serum (FBS), as well as in a serum-free culture medium.

**Figure 1:**
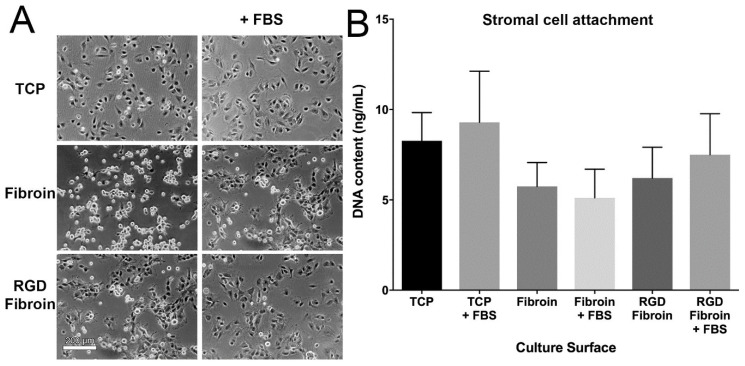
(**A**) Visual comparison of limbal stromal cell attachment to TCP, TCP coated with Fibroin (BMSF), or TCP coated with RGD-Fibroin. Cells were seeded at a density of 15,000 cells/cm² in 24-well culture plates and incubated for 90 min in the absence or presence of FBS in the culture medium. Phase contrast images display the typical appearance of cells after 90 min incubation followed by rinsing in phosphate-buffered saline. (**B**) Quantification of stromal cell attachment via measurement of dsDNA content using the PicoGreen assay. Bars represent the mean ± SEM for cultures derived from 5 unique donors. No significant difference between culture surfaces/conditions was detected (Friedman test with Dunn’s multiple comparisons test; *n* = 5).

Visual assessment by phase contrast microscopy revealed that while similar numbers of stromal cells appeared to be present under all conditions tested, the majority of cells that had attached to non-modified fibroin under serum-free conditions were noticeably less spread than those in any other wells (Figure 1). In contrast, stromal cells seeded onto RGD-fibroin in the absence of serum displayed a similar degree of spreading compared to those grown on either fibroin surface in the presence of serum. Nevertheless, quantification of dsDNA content within each well (for five unique tissue donors, with each surface being tested in quadruplicate for each donor) indicated a similar number of cells attachment to each well irrespectively of coating or presence of serum. On this basis, it was concluded that despite apparent effects on the cell shape (i.e., spreading) in the absence of serum, the RGD-fibroin, did not appear to offer significant benefits to stromal cell attachment in a growth medium supplemented with serum.

#### 2.1.2. Attachment of Limbal Epithelial Cells

**Figure 2:**
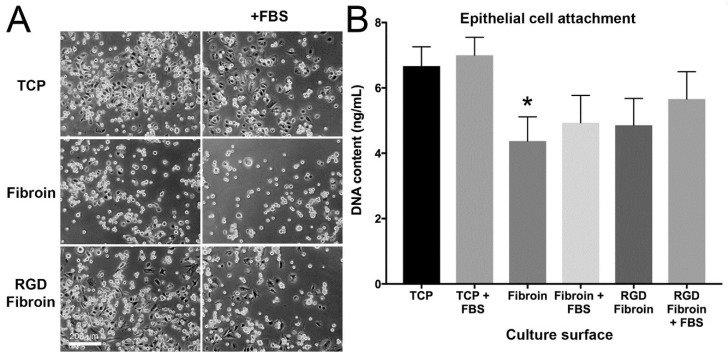
(**A**) Visual comparison of limbal epithelial cell attachment TCP, TCP coated with Fibroin (BMSF), or TCP coated with RGD-Fibroin. Cells were seeded at a density of 25,000 cells/cm² in 24-well culture plates and incubated for 90 min in the absence or presence of 10% (*v*/*v*) FBS in the culture medium. Phase contrast images display the typical appearance of cells after 90 min incubation followed by three rinses in phosphate-buffered saline. (**B**) Quantification of epithelial cell attachment via measurement of dsDNA content using the PicoGreen assay. Bars represent culture data derived from 4 unique donors. Asterisk indicates a significant difference (*p* < 0.05) between Fibroin-coated TCP compared to TCP in serum-supplemented culture medium (+FBS) (Friedman test with Dunn’s multiple comparisons test; *n* = 4).

The relative attachment of limbal epithelial (LE) cells to fibroin-coated TPC was evaluated in same way as for the limbal stromal cells. Visual assessment of LE cell morphology indicated that these cells were also noticeably less spread after 90 min incubation on standard fibroin (BMSF) compared to RGD-fibroin (Figure 2). Unlike with stromal cells, however, the presence of serum appeared to have little impact on the spreading of LE cells attached to regular fibroin. Quantification of dsDNA content within each well, for 4 donors of LE cells, with each surface being tested in quadruplicate for each donor, confirmed that similar numbers of cells had attached to each surface, with the exception of fibroin under serum-free conditions that supported the attachment of significantly less cells than for the positive control (TCP) in the presence of serum (Figure 2). Thus, as for stromal cells, the practical benefits of using RGD-fibroin over standard fibroin as a short-term substrate for LE cells appeared to be negligible with respect to encouraging cell attachment.

### 2.2. Long-Term Response of Limbal Cells to RGD-Fibroin

#### 2.2.1. Growth Behaviour of Limbal Stromal Cells

**Figure 3:**
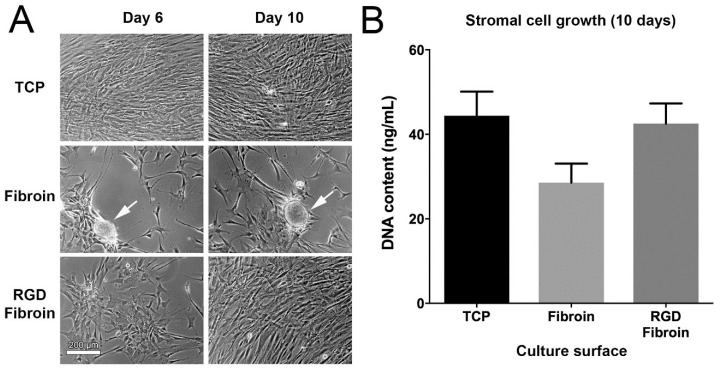
(**A**) Visual comparison of limbal stromal cell growth behaviour in the presence of serum (10% *v*/*v* FBS) on TCP, TCP coated with Fibroin (BMSF), or TCP coated with RGD-Fibroin. Cells were seeded at a density of 15,000 cells/cm² in 24-well culture plates and photographed after 6 and 10 days, respectively. White arrows indicate the presence of cell clumps that became more apparent in cultures established on Fibroin-coated TCP. (**B**) Quantification of stromal cell growth after 10 days using PicoGreen assay. Bars represent data obtained using stromal cell cultures established from 4 unique donors. Despite clumping, no significant differences in cell numbers (as measured by dsDNA content) were observed after 10 days (Friedman test with Dunn’s multiple comparisons test).

For long-term studies, fibroin-coated TCP was once again used as a control. Visual assessment of cultures after 6 days in complete culture medium (containing 10% FBS) demonstrated that while the stromal cells had grown to confluency on TCP, the cultures established at the same seeding density on either formulation of fibroin were approximately only 20–40% confluent (Figure 3). By day 10, however, the confluency of stromal cells on RGD-fibroin resembled more closely that observed on TCP, with far fewer clusters. In contrast, stromal cells grown for 10 days on non-modified fibroin remained sub-confluent, and aggregated clumps of cells became more apparent. Nevertheless, quantification of the dsDNA content within each well (for four unique tissue donors, with each surface being tested in quadruplicate for each donor) once again indicated no significant difference in cells numbers between the culture conditions examined. Thus, while the presence of RGD motifs had no practical effect on overall cell numbers, it significantly reduced the clumping of stromal cells observed for cultures grown on regular fibroin when grown in the presence of serum. On this basis, it was concluded that there are practical benefits to using RGD-fibroin in order to achieve a more even coverage of stromal cells.

#### 2.2.2. Stratification of Limbal Epithelial Cells on Freestanding Membranes

**Figure 4:**
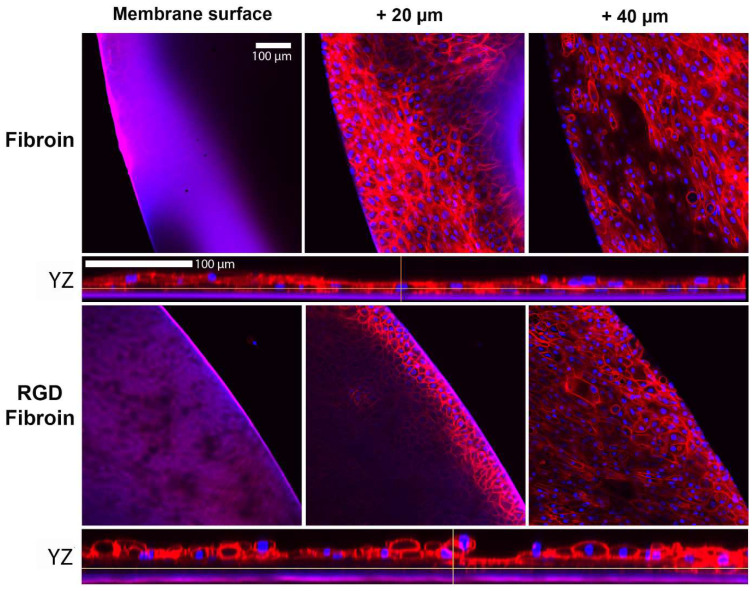
Evaluation of limbal epithelial cell growth (stratification) on freestanding fibroin membranes. Confocal fluorescence micrographs demonstrate the relative thickness and confluence of basal and stratified layers for limbal epithelial cells grown on Fibroin (BMSF) membranes, compared to membranes prepared from RGD-Fibroin. Cultures were maintained in serum-supplemented growth medium for approximately 2 weeks prior to fixation in neutral buffered formalin and labelling with rhodamine phalloidin and Hoechst nuclear stain. A tighter and enhanced cobblestoned morphology is observed for the culture grown on RGD-Fibroin (as displayed at approximately +20 µm above the plane of the membrane surface). The corresponding enlarged YZ axis view is displayed underneath each set of images. The cultures grown on RGD-Fibroin membranes were also typically more stratified as demonstrated by greater confluency at +40 µm above the plane of the membrane and the overall number of layers produced (2–4 layers compared to 1–2 layers).

Given the positive results for long-term cultures of stromal cells grown on RGD-fibroin, we proceeded to manufacture freestanding membranes from this material. The resulting membranes were subsequently mounted into specialized cell culture chambers that support the long-term culture and stratification of corneal limbal epithelial cells by cultivation at the air-liquid interface [24]. After 2 weeks in culture, the degree of stratification was compared to the cultures grown on standard fibroin membranes by fixation using formalin and subsequent staining with rhodamine phalloidin, a probe that demonstrates the F-actin network within cells. Through the use of confocal fluorescence microscopy (Figure 4), it became evident that while confluency was achieved on both membranes, the cultures established on RGD-fibroin displayed a denser cobblestone-like morphology within their basal layer (e.g., when imaged approximately 20 µm above membrane surface) compared to those grown on conventional fibroin membranes. Moreover, when scanning towards the surface of each culture (e.g., at 40 µm above membrane surface), it became apparent that cultures grown on RGD-fibroin displayed a greater number of confluent layers. Based upon this result, we proceeded to optimize the mechanical and permeability characteristics of RGD-fibroin membranes through the use of PEG (as a porogen) and HRP (as a crosslinking agent).

### 2.3. Mechanical Properties of Freestanding RGD-Fibroin Membranes

**Figure 5:**
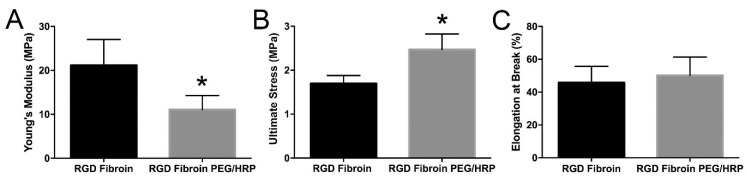
Evaluation of mechanical properties for freestanding RGD-Fibroin membranes when cast either with or without use of PEG as a porogen and the enzyme HRP as a crosslinking agent. (**A**) Young’s modulus, (**B**) Ultimate stress, and (**C**) Percentage elongation at break. All values are mean ± standard deviation of six measurements. Asterisk indicates significantly different value to RGD-Fibroin (*p* < 0.01; non-paired two-tailed *t* test).

The RGD-fibroin membranes treated with PEG and HRP were approximately 3-fold thicker and more rigid than the original membranes. The modified membranes were easier to handle when wet but became noticeably more brittle when dried than the untreated RGD-fibroin membranes. Hydrated RGD-PEG/HRP-fibroin membranes demonstrated a lower Young’s modulus compared to the RGD-fibroin membranes (11.1 ± 3.2 vs. 21.2 ± 5.8 MPa; *p* < 0.01; Figure 5A), while ultimate stress was found to be significantly higher (2.5 ± 0.4 vs. 1.7 ± 0.2 MPa; *p* < 0.01; Figure 5B). The values for elongation at break for each membrane type were similar (Figure 5C).

### 2.4. Permeability Characteristics of Freestanding RGD-Fibroin Membranes

**Figure 6:**
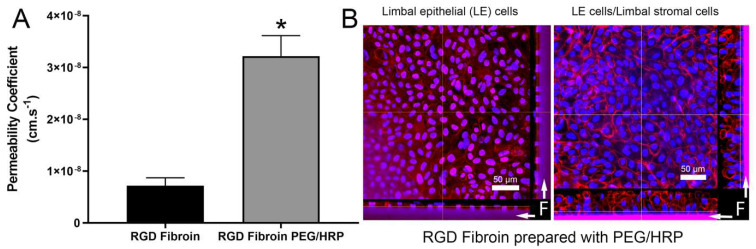
Permeability characteristics of RGD-Fibroin membranes. (**A**) Relative permeability of RGD-Fibroin compared to RGD-PEG/HRP-Fibroin examined by diffusion of Allura Red AC (MW 496.4 Da). Bars represent mean ± standard deviation from four separate measures. Asterisk indicates significantly different value to RGD-Fibroin (*p* < 0.0001; non-paired two-tailed *t* test). (**B**) Further evidence of membrane permeability is provided by demonstrating increased stratification (confocal microscopy) of limbal epithelial cell cultures (LE) when grown on RGD-PEG/HRP-Fibroin membranes in the absence and presence of limbal stromal cells (cultivated on the opposing membrane surface; beyond field of view imaged). Parallel cultures prepared from the same donor’s LE cells were maintained for 12 days prior to fixation and staining with rhodamine phalloidin (red/pink) and Hoechst nuclear stain (blue). Fibroin membrane (labelled “F” with arrows) is visible within the Z profile views (at the bottom and right-hand side of each image) due to a combination of autofluorescence and some residual excess rhodamine phalloidin.

Permeability to Allura Red AC dye (MW 496.4 Da) was approximately 4-fold higher (*p* < 0.0001) for the RGD-PEG/HRP-fibroin membranes compared to the RGD-fibroin membranes (Figure 6A). Given the relatively low molecular weight of the dye used, a more biologically relevant demonstration of permeability was attempted by examining the effect of a culture of limbal stromal cells on the stratification of limbal epithelial cells, when the two cell types are grown on opposing surfaces of the RGD-PEG/HRP-fibroin membranes. Importantly, the design of the cell culture chambers used [24] supports separation of the upper and lower culture compartments such that communication between the two cell types is restricted to what permeates through the membrane. The influence of stromal cells on epithelial stratification was subsequently examined by confocal fluorescence microscopy. Staining with rhodamine phalloidin demonstrated that epithelial cultures grown in the presence of an underlying layer of limbal stromal cells were 2 to 4-fold more stratified than those grown in the absence of stromal cells (Figure 6B). This finding suggests that the PEG/HRP–RGD–fibroin membranes support the communication of stromal cells and epithelial cells via diffusion of soluble factors.

## 3. Discussion

While the suitability of silk fibroin membranes as substrates for growing various types of corneal cells has been well established [2,5,11,12,13,14,17,18,19,22,25], an optimal formulation has yet to be determined for clinical use. If compared to the current “gold” standard for corneal epithelial cell transplantation, the human amniotic membrane, an optimal formulation should support long-term cell attachment and be sufficiently permeable to support the diffusion of nutrients and epithelial–stromal cell communication. To address this issue, the present study has explored the potential of fibroin isolated from silkworm cocoons that have been genetically modified to incorporate the cell-binding RGD motif. In addition, treatments with PEG and HRP have been used as means for optimizing the permeability and strength of the resulting RGD-fibroin membranes, respectively. The resulting membranes display a number of positive qualities that make them an attractive candidate for further study.

With respect to cell attachment, we had anticipated that RGD-fibroin would encourage a greater number of cells to adhere than that observed on BMSF. This hypothesis was based upon an extensive body of literature establishing the role of the cell-binding RGD motif [26,27], and especially the encouraging data obtained for the proliferation of chondrocytes on RGD-fibroin [20,21]. Moreover, enhanced growth of a transformed human corneal fibroblast cell line has been reported [18] when cultured on BMSF membranes labelled with the RGD motif. We were, therefore, initially surprised to observe that the RGD-fibroin had no practical benefits on the attachment of either corneal cell type over 90 min. Nevertheless, others [18] did not observe any differences in dsDNA content of cultures within the first 24 h. In another study [21], the differences in adhesive force (rather than dsDNA) were quantified and their earliest measurements were made 3 h after the seeding of chondrocytes. Thus, our results for corneal stromal cell attachment are actually consistent with those in a previous report [18], but differ from other reported results [21], likely owing to differences in the timing of observations, parameter measured, and cell type. In any case, our subsequent analyses of longer-term cultures provide more compelling evidence of positive effects for RGD-fibroin on corneal cell attachment and growth.

At this stage of discussion, it is worth to mention that there is evidence that the simple presence of RGD motifs on a material surface is not enough for imparting cell-adhesive characteristics to the material [26,28]. Although the RGD sequence is a rather ubiquitous ligand that is recognized by a plethora of integrin receptors, there are certain critical factors governing the efficacy of a cell-to-surface adhesion event [26,29,30,31,32], including surface density and spatial distribution of RGD motifs, nature of the RGD-containing precursor peptide, and presence and length of spacers involved in the presentation to cells of the RGD sequence. Investigating such factors was beyond the purpose of this study.

The clumping of stromal cells cultures observed when grown for several days on fibroin membranes is similar to that reported previously by our group for retinal pigment epithelial cells [15]. In each case, we have interpreted the detachment and contraction of cultures over time as resulting from a difference in the greater relative strength of cell–cell adhesiveness compared to cell-fibroin adhesion. Moreover, the cells may display a preferred adherence to the ECM components that they secrete, which in addition, may lack sufficient tethering in an even manner to the underlying fibroin membrane. In the case of RPE cultures, which require several weeks for maturation, improved adherence of our cultured monolayers was achieved via pre-coating of the fibroin membrane with collagen [15]. It, therefore, seems likely that the reduced clumping of corneal stromal cell cultures observed presently was due to the effect of RGD sequences distributed across the surface of the fibroin films. Nevertheless, further studies including inhibition throughout addition of soluble RGD peptide are required to confirm this.

Given our prior results with RPE cells [15] and the present observations with corneal stromal cells, it was interesting to note that our present long-term cultures of corneal limbal epithelial cells seemed less prone to clumping over time. A potential explanation for this difference is that these cells tend to form stratified layers and secrete different ECM molecules in the form of basement membrane components. Moreover, prior analyses of stromal cultures similar to those in this study contain significant number of myofibroblasts and therefore are more contractile than epithelial cells cultures [33]. As such, RGD-fibroin did not seem to significantly benefit the short-term culture of corneal limbal epithelial cells beyond some minor effects on cell spreading. Nevertheless, the altered morphology for stratified epithelial cultures observed on RGD-fibroin (Figure 4) provides evidence that the basal layer of cells was potentially better anchored, thus facilitating stratification of the cultures. Future studies should therefore examine the relative expression and distribution of the different cytokeratin proteins associated with basal cell attachment (e.g., cytokeratin-5, -14, and -15) and stratifying corneal epithelium (cytokeratin-3 and -12). Moreover, it remains to be seen what influence RGD-fibroin has on the expression of integrin proteins by both cell types examined in this study.

With regard to permeability, PEG has been frequently used as a tool for enhancing this characteristic in fibroin films and membranes [22,34]. During casting and subsequent drying, the PEG molecules coalesce into hydrophilic regions that are subsequently removed upon washing in water. While PEGs of higher molecular weight (>20 kDa, commonly known as poly(ethylene oxides)) can be used to promote the formation of larger pores, the inclusion of lower molecular weight PEGs (e.g., 300 Da) results in increased permeability as measured by the movement of various substances (gases, dyes, polymers, proteins). As the freestanding fibroin membranes prepared with PEG were too fragile to support clinical applications, the crosslinking with the plant-derived and non-toxic substance genipin has been applied to increase the stability of membranes [22]. In the present study, the enzyme horseradish peroxidase was used as a crosslinking agent since it proved to be more effective than genipin [23]. The combined treatments resulted in freestanding membranes that, while thicker, were flatter and easier to handle than the RGD-fibroin membranes. Moreover, the resulting membranes remained sufficiently permeable to support increased stratification in response to an underlying layer of limbal stromal cells (Figure 6).

## 4. Materials and Methods

### 4.1. Sourcing of Fibroin

Standard fibroin (BMSF) was sourced as *Bombyx mori* silk cocoons supplied by Tajima Shoji Co. Ltd. (Yokohama, Japan), all cut in half and with the pupae removed. Approximately 2.5 g of cut cocoon pieces (~1 cm^2^ in size) were kept in 1 L boiling solution of sodium carbonate (0.02 M) for 1 h to remove sericin (i.e., the degumming step). The degummed fibers were subsequently washed in 1 L of water at 60 °C for 20 min, three times in succession with intermittent squeezing to remove the excess liquid. The fibers were then dried in a fume hood for at least 12 h.

The recombinant RGD-fibroin, as originally described [20,21], was provided for this study as degummed fibers by the National Agriculture and Food Research Organization (NARO, Tsukuba, Japan).

### 4.2. Preparation of Fibroin Solutions from Degummed Fibers

Dried silk fibers containing either standard fibroin (BMSF) or RGD-fibroin were dissolved in a 9.3 M lithium bromide aqueous solution at 60 °C for 4 h, and the resulting solution was transferred into a dialysis cassette (MWCO at 3.5 kDa) and dialyzed for 3 days with 6 water exchanges throughout. Each fibroin solution was filtered through two successive syringe filters (porosities of 0.7 and 0.2 µm, respectively), and stored at 4 °C. The resulting solutions, with a concentration of about 3% *w*/*v* fibroin (as determined by gravimetric analysis) were diluted with water to the required concentration.

### 4.3. Coating of Tissue Culture Plastic with Fibroin

Fibroin solution was dispensed into a 24-well tissue culture plate to create fibroin coatings (using 2% *w*/*v* fibroin solution, 256 µL/well). After drying at room temperature, the fibroin-coated plates were water annealed in a vacuum chamber at −80 kPa for 6 h at room temperature in the presence of a container filled with water in the chamber. Wells were sterilized by applying 70% ethanol for 1 h followed by one rinse with phosphate-buffered saline (PBS) and 2 rinses with serum-free culture medium.

### 4.4. Preparation of Freestanding Fibroin Membranes

Freestanding fibroin membranes were prepared by casting a 1.78% *w*/*v* fibroin solution onto a custom-made casting table where the supporting glass plate was pre-coated with a polyolefin polymer (Topas^®^) film. The blade height was set in order to generate an approximate dry thickness of 6 μm for the resulting fibroin membranes. After drying at room temperature, the membranes were water-annealed in a vacuum chamber at −80 kPa for 6 h at room temperature in the presence of a container filled with water. The freestanding membranes were then peeled off from the supporting Topas^®^ film, and subsequently sterilized by applying 70% ethanol for 1 h followed by one rinse with PBS and two rinses with serum-free culture medium.

### 4.5. Optimization of Freestanding Fibroin Membranes Using PEG and HRP

PEG was introduced as a porogen to increase membrane permeability as has been established elsewhere [22,27]. Since this treatment makes the membranes mechanically weaker [22], the HRP-induced crosslinking of fibroin (through the formation of dityrosine linkages [23]) was performed to increase their strength. Stock solutions of HRP (150 U/mL) and hydrogen peroxide (H_2_O_2_) (0.3%) were first prepared. Then, PEG was slowly blended into the 1.78% RGD-fibroin solution at a PEG/fibroin ratio of 2:1 (by weight), followed by the addition of equal volumes of HRP and H_2_O_2_ solutions. The final concentration of HRP in the mixture was 1.1 U for 1 mg of protein (RGD-fibroin). The mixture was then cast into a Topas^®^ pre-coated Petri dish covered with a lid and stored at 40 °C for 2 h to form a gel. The volume was set to obtain 1.81 mg fibroin/cm^2^. The resulting gel was dried in a fan-driven oven at room temperature for at least 12 h. After drying, the membranes were soaked in water (1 L/dish) for 3 days with two water exchanges per day to remove the PEG. The membranes were subsequently treated with 3% H_2_O_2_ solution for 10 min at room temperature to quench any residual HRP activity, followed by rinsing with water three times. The membranes were peeled off from the underlying Topas^®^ film and stored in water at 4 °C.

### 4.6. Mechanical and Permeability Testing of Fibroin Membranes

Membrane strips (1 × 3 cm) were subjected to tensile measurements in an Instron Materials Testing System Model #5943 (Instron, Norwood, MA, USA), equipped with a 50-N load cell and a set gauge distance and crosshead speed of 14 mm and 14 mm/min, respectively. The samples were loaded with pneumatic grips and submerged in PBS, and pre-heated to 37 °C ± 3 °C in a BioPuls™ unit for 5 min prior to stretching. Stress–strain curves were plotted, the elongation and ultimate stress at break were recorded, and the Young’s moduli were computed in the linear region. The mean values were calculated from results generated by 6 measurements for each specimen. Permeability tests using Allura Red AC dye were conducted as detailed elsewhere [16].

### 4.7. Sourcing of Human Tissue

Samples of cadaveric human corneal limbus (up to approximately 10 days post-mortem) stored in Optisol corneal preservation medium, were obtained in the form of corneoscleral rims discarded following routine corneal transplant surgeries performed at the Queensland Eye Hospital, Brisbane, Australia. All tissue used had previously been consented for research during the process of procurement through the Queensland Tissue Bank, Brisbane, Australia.

### 4.8. Isolation and Culture of Limbal Stromal Cells

The tissue samples were washed twice in Hanks’ balanced salt solution (HBSS), incubated in 2.5 mg/mL Dispase II (Gibco Cat. No. 17105-041) for 1.5 h at 37 °C and scraped with a scalpel blade to remove the epithelial and endothelial tissue layers. A trephine blade (2 mm diameter) was then used to obtain several punch biopsies of limbal stroma. The biopsies were attached to the bottom of tissue culture dishes using 30 µL of type I collagen gel (1 mg/mL) and subsequently submerged in stromal cell growth medium consisting of Dulbecco’s modified Eagle’s medium (DMEM) with high glucose content (Life Technologies Cat. No. 10313-021), 10% (*v*/*v*) foetal bovine serum (FBS), 2 mM L-glutamine (Life Technologies Cat. No. 25030-081) and 1% penicillin/streptomycin solution (Life Technologies Cat. No. 15140-122). The resulting primary culture was subsequently harvested by rinsing with Versene (Gibco Cat. No. 15040066) followed by incubation for 5–10 min in TrypLE select enzyme (Gibco Cat. No. 12563011). Cultured were expanded to passage p2 before re-suspension in 90% FBS/10% dimethyl sulfoxide (DMSO) and storage, at 2 × 10^6^/mL in liquid nitrogen.

### 4.9. Isolation and Culture of Limbal Epithelial Cells

After washing in HBSS, the tissue samples were cut into quarters, trimmed using a scalpel, and digested for 1 h in 2.5 mg/mL Dispase II dissolved in DMEM. Epithelial cells were subsequently harvested from the limbal margin of the cornea by scraping and aspirating with a pipette tip. The harvested epithelial cells were subsequently washed and re-suspended in epithelial cell growth medium (with centrifugation at 300× *g* for 5 min) before being seeded into a 25 cm^2^ culture flask pre-seeded with 10^6^ growth-arrested (using 2 × 25 Gy) murine 3T3 cells (ATCC; CCL92). The epithelial culture medium consisted of DMEM combined in a 3:1 ratio with Hams F12 (Life Technologies 11765-062, Carlsbad, CA, USA) and supplemented with 10% FBS (HyClone, SH 30084.03, Australia), 2 mM L-glutamine, 10 ng/mL recombinant human epidermal growth factor (EGF; Invitrogen PHG0311, San Diego, CA, USA), 5.6 µg/mL isoproterenol (Sigma Cat. No. I6504, St. Louis, MO, USA), 180 µg/mL adenine hydrochloride hydrate (Sigma Cat. No. A9795), 5 µg/mL transferrin (Sigma Cat. No. T1147, St. Louis, MO, USA), 1% non-essential amino acids (Life Technologies Cat. No. 11140-050), 1.36 ng/mL tri-iodo-L-thyronine sodium salt (T3) (Sigma-Aldrich Cat. No. T6397), 1 µg/mL insulin (Sigma Cat. No. I6634), 0.4 µg/mL hydrocortisone (Sigma Cat. No. H4001) and 1% penicillin/streptomycin solution (Life Technologies Cat. No. 15140-122). Cultures were passaged as described above for stromal cells and typically tested at passage p2.

### 4.10. Cell Attachment Assay

Cells were applied to culture surfaces at a density of either 15,000 cells/cm² (stromal cells) or 25,000 cells/cm² (epithelial cells) and incubated for 90 min prior to analysis. The subsequent morphology and numbers of attached cells was examined under both serum-free and serum-supplemented growth conditions. Test culture surfaces included TCP, TCP coated with standard fibroin (BMSF) and TCP coated with RGD-fibroin. After 90 min, the cultures were rinsed briefly 3 times with HBSS to remove any non-attached cells. Photographs of each culture were taken using an Olympus TS-100 inverted phase contrast microscope equipped with a 10× objective lens. Following photography, the buffer was removed, and each plate stored frozen at −80 °C until analysis of dsDNA content in each well using the Quant-iT™ PicoGreen^®^ dsDNA assay (kit, Invitrogen Cat. No. P11496), according to manufacturer’s instructions and as detailed in a previous report [17]. For long-term (up to 10 days) examination of stromal cell growth, the cultures were maintained in serum-supplemented growth medium until analysis.

### 4.11. Establishment of Cultures on Freestanding Fibroin Membranes

Membranes fabricated from either BMSF, RGD-fibroin or RGD-PEG/HRP-fibroin were mounted in custom designed cell culture chambers [24]. For initial long-term studies of epithelial cell growth, cells were seeded as at a density of 10^5^ cells/cm² and cultured for 10–12 days. The volume of cell culture medium was reduced after ~1 week to encourage epithelial stratification at the air-liquid interface. For later co-culture experiments, the membranes were initially seeded with stromal cells at a density of 0.5 × 10^5^ cells/cm² on one side of each membrane. After 2–3 days, the chamber was inverted, and epithelial cells seeded onto the opposite side as described above. The cultures were subsequently maintained in epithelial growth medium. The resulting cultures were grown for ~12 days prior to fixation and staining for analysis by confocal microscopy.

### 4.12. Confocal Analysis of Epithelial Growth and Stratification

For the analysis of structure, the co-cultures were fixed in 10% buffered formalin and subsequently stored in PBS. A 4 mm trephine punch was subsequently used to sample cultures for further analysis. Samples were treated with 0.1% Triton X-100 in PBS for 30 min before staining with rhodamine phalloidin (1:100 dilution in PBS) and 1 µM Hoechst 33342 overnight at 4 °C. The stained samples were subsequently washed extensively in fresh PBS (3–4 washes of 30 min each) and mounted for confocal microscopy under glass coverslips in a 1:1 mixture of PBS and glycerol. A Nikon A1 confocal system equipped with a Plan Apo 20×/0.75 N.A. lens was used to construct stacks of approximately 45–50 XY images, with a Z-step size of between 0.5 and 1 µm and the pinhole set to ~1 airy unit.

### 4.13. Statistical Analysis

Statistical analyses were conducted using GraphPad^®^ Prism software. Based upon the results of initial tests for normality, data was subsequently analysed using either a non-parametric ANOVA (Friedman test with Dunn’s multiple comparisons test; for cell attachment data) or a non-paired two-tailed *t* test (for tests of mechanical properties and permeability).

## 5. Conclusions

The recombinant RGD-fibroin developed by Kambe et al. [20,21] displays advantages for the cultivation of corneal cells when compared to the non-modified fibroin (BMSF). The main advantage for corneal stromal cell culture is to facilitate more even growth across the surface of fibroin membranes, as opposed to forming aggregated clumps of cells. The main advantage for epithelial cell culture is to encourage stratification, thus better mimicking the desired properties of the corneal epithelium observed in vivo. Moreover, the permeability and handling characteristics of freestanding membranes can be optimized through the use of PEG (as a porogen) and HRP (as a crosslinking agent). Further studies are required to confirm that the benefits of recombinant RGD-fibroin are due to the presence of RGD motifs. It also remains to be investigated whether the observed benefits are linked to alterations in integrin expression, ECM molecules deposition or events mediated at the transcriptional level.

## Data Availability

The authors can confirm that all relevant data are included in this published article. If needed, the raw data associated with the quantitative findings of this study will be made available upon written request to the author D.G.H.

## Sample Availability

Samples of the compounds employed in this study are not available from the authors.
